# Histone deacetylase inhibitor sodium butyrate suppresses DNA double strand break repair induced by etoposide more effectively in MCF-7 cells than in HEK293 cells

**DOI:** 10.1186/s12858-014-0030-5

**Published:** 2015-01-16

**Authors:** Liping Li, Youxiang Sun, Jiangqin Liu, Xiaodan Wu, Lijun Chen, Li Ma, Pengfei Wu

**Affiliations:** Key Laboratory for Medical Molecular Diagnostics of Guangdong Province, Guangdong Medical College, Xincheng Road, Dongguan, 523808 P R China; Department of Biochemistry, School of Basic Medicine, Guangdong Medical College, Dongguan, 523808 P R China

**Keywords:** Double strand breaks, Histone deacetylase inhibitor, MCF-7, HEK293, Etoposide, Sodium butyrate

## Abstract

**Background:**

Histone deacetylase inhibitors (HDACi’s) are emerging as promising anticancer drugs alone or in combination with chemotherapy or radiotherapy agents. Previous research suggests that HDACi’s have a high degree of selectivity for killing cancer cells, but little is known regarding the impact of different cellular contexts on HDACi treatment. It is likely that the molecular mechanisms of HDACi’s involve processes that depend on the chromatin template, such as DNA damage and repair. We sought to establish the connection between the HDACi sodium butyrate and DNA double-strand break (DSB) damage in human breast cancer MCF-7 and non-cancerous human embryonic kidney293 (HEK293) cells.

**Results:**

Sodium butyrate inhibited the proliferation of both HEK293 and MCF-7 cells in a dose- and time- dependent manner, but the effects on MCF-7 cells were more obvious. This differential effect on cell growth was not explained by differences in cell cycle arrest, as sodium butyrate caused an arrest in G_1_/G_2_ phase and a decrease in S phase for both cell lines. At high doses of sodium butyrate or in combination with etoposide, MCF-7 cells formed fewer colonies than HEK293 cells. Furthermore, sodium butyrate enhanced the formation of etoposide-induced γ-H2AX foci to a greater extent in MCF-7 than in HEK293 cells. The two cells also displayed differential patterns in the nuclear expression of DNA DSB repair proteins, which could, in part, explain the cytotoxic effects of sodium butyrate.

**Conclusions:**

These studies suggest that sodium butyrate treatment leads to a different degree of chromatin relaxation in HEK293 and cancerous MCF-7 cells, which results in differential sensitivity to the toxic effects of etoposide in controlling damaged DNA repair.

## Background

Eukaryotic DNA is bound by histones and organized into chromatin, which serves as the true in vivo substrate of transcription, replication and DNA repair. Post-translational modification of histones alters chromatin structure; for example, histone acetylation plays a central role in the unwinding of DNA. Histone deacetylase inhibitors (HDACi’s) globally increase histone acetylation, relaxing chromatin structure and leading to reversible decondensation of chromatin regions [1]. These inhibitors of chromatin-modifying enzymes are emerging as a promising anticancer drug and already have shown anticancer effects in both pre-clinical and clinical settings [2,3]. HDACi’s are gaining increasing attention because of their therapeutic effectiveness in selectively killing cancer cells and their mild toxicity profile [3-5].

Double strand breaks (DSBs) in DNA occur naturally in the genome during replication and are increased by exogenous DNA damaging agents. Many anti-cancer therapeutics, including radiotherapy and chemotherapy agents, kill tumor cells by inducing DSBs. DSB repair is essential for cell viability and normal growth because a single unrepaired DSB can lead to programmed cell death. DSBs can be repaired through several pathways including homologous recombination, non-homologous end-joining and single strand annealing [6]. Understanding the relationship between DSB repair and HDACi anticancer effects is important for elucidating mechanistic details of DSB repair within chromatin that have the potential to be exploited in the clinic.

Sodium butyrate, a naturally occurring short-chain fatty acid that is a by-product of carbohydrate metabolism in the gut, is one of the most widely studied HDACi’s [7]. We studied the effect of sodium butyrate alone and combination with the DNA damaging agent, etoposide. Etoposide, a classical chemotherapeutic drug of cancer, interrupts the normal function of topoisomerase II (Topo II) during DNA replication and generates DSBs [1]. We treated both healthy human embryonic kidney 293 (HEK293) and breast cancer MCF-7 cells with sodium butyrate, and our results demonstrate that sodium butyrate treatment increases sensitivity to the cytotoxic effect of etoposide and reduces DSB repair capacity in MCF-7 but not in HEK293 cells.

## Methods

### Ethics statement

All results of this research were based on the use of cultured human (MCF-7) cell lines. Neither human (human subjects or human derived material) nor animals (vertebrates or any regulated invertebrates) were used in this experimental research.

### Cell lines and reagents

Human breast cancer cell MCF-7 and human embryonic kidney 293 cells were obtained from Dr. Fen Xia (The Ohio State University College of Medicine, Columbus, Ohio.). The cells were maintained in DMEM supplemented with 10% fetal bovine serum, 50 units/mL penicillin, and 50 μg/mL streptomycin (Invitrogen, Gibco) at 37°C under 5% CO_2_.

Sodium butyrate was purchased from Sigma-Aldrich, and etoposide from Selleck Chemicals. The subcellular protein fraction kit was purchased from Thermo Scientific, and the Cell Counting Kit (CCK-8) was from Beyotime (P.R. China). Rabbit antibodies for Rad51, Rad52 and CtIP were purchased from Abcom, and rabbit antibodies for Ku80 and RPA70, mouse antibodies for acetyl histone H4 and γ-H2AX, and secondary antibody for anti- rabbit, anti-mouse and Alexa-fluor488-conjugated anti-mouse were purchased from Cell Signaling Technology. All other reagents were of analytic grade and purchased from standard suppliers.

### Cell proliferation assays

HEK293 and MCF-7 cells were seeded in a 96-well plate at a density of 3×10^3^ cells/well and then were treated with DMSO vehicle or various concentrations of sodium butyrate in 100 μl medium for the indicated times. After the treatment period, 10 μl CCK-8 mixture was added to each well, and the plates were incubated for 40 min at 37°C. The absorbance was measured in a microplate reader (Biotek, USA) at a wavelength of 450 nm.

### Cell cycle analysis

HEK293 and MCF-7 cells were treated with either DMSO vehicle or various concentrations of sodium butyrate for 24 h, and then cell cycle distribution was determined using standard ethanol fixation and propidium iodide staining followed by flow cytometry (Bio-Rad, USA) as previously described [8].

### Colony forming assay

HEK293 and MCF-7 cells were treated with either DMSO vehicle or various concentrations of sodium butyrate for 4 h followed by the addition of DMSO vehicle or etoposide to a final concentration of 0.5 μM for another 20 h incubation. After the treatment period, cells were counted and reseeded in duplicate in 60 mm dishes at a density of 300 cells per dish. Colonies were stained after 15 d incubation and counted as positive if >50 cells were visible. The survival fraction was calculated as follows: (number of colonies for sodium butyrate/number of cells plated)/(number of colonies for corresponding control/number of cells plated).

### Immunofluorescence assay

Immunohistochemistry was performed as previously described [8]. Staining patterns were visualized via fluorescence microscopy (Leica, German). Cells with >5 foci were recorded, and100 to 300 cells were counted per slide. The average foci in one cell and the percentage of foci-positive cells in the whole cell population were calculated.

### Western blot analysis

Soluble nuclear and chromatin-bound protein fractions were extracted using a subcellular protein fraction kit. 50 μg protein per well were separated by SDS/polyacrylamide gel electrophoresis and electroblotted onto nitrocellulose membranes. The transblotted membranes were washed twice with TBS containing 0.1% Tween20 (TBST). After blocking with TBST containing 5% nonfat milk for 40 min, the membranes were incubated with an appropriate primary antibody in TBST containing 1% nonfat milk at 4°C overnight. After treatment with the primary antibodies, the membranes were washed twice with TBST for a total of 20 min, followed by incubation with anti-rabbit or anti-mouse IgG-HRP conjugates for 1 h at room temperature and then washed twice with TBST for a total of 1 h. The immunoblots were visualized by enhanced chemiluminescence.

### Statistical analysis

The data were analyzed via ANOVA followed by a Bonferroni post test using GraphPad Prism version 4.02 for Windows (GraphPad Software).

## Results

### Sodium butyrate suppresses the proliferation of HEK293 and MCF-7 cells to different extents

**Figure 1:**
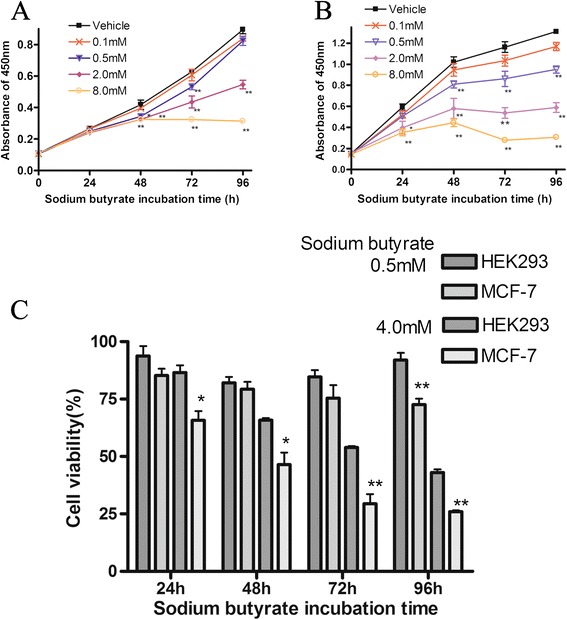
**Sodium butyrate suppresses the proliferation of HEK293 and MCF-7 cells to different extents. (A)** HEK293 cells were incubated with DMSO vehicle, 0.1, 0.5, 2.0, or 8.0 mM sodium butyrate for the indicated times. After the treatment period, cells were assessed for cell proliferation by CCK-8 assay. The A_450nm_ value corresponds to the amount of cells. *P< 0.05 and **P< 0.01 versus the corresponding time for vehicle-treated cells. **(B)** MCF-7 cells were incubated with DMSO vehicle or sodium butyrate and were assessed by CCK-8 assay as in panel A. **(C)** MCF-7 cell growth inhibition was compared for HEK293 versus MCF-7 cells after treatment with 0.5 or 4.0 mM sodium butyrate for the indicated times. Results represent the CCK-8 assay values at each respective drug treatment relative to that of the DMSO vehicle control. *P< 0.05 and **P< 0.01 for MCF-7 cells versus the corresponding treatment for HEK293 cells. All data represent the means +/− SD of 3 experiments performed in triplicate.

To verify the effects of sodium butyrate on the proliferation of HEK293 cells and cancerous MCF-7 cells, we performed CCK-8 assays. Sodium butyrate suppressed both HEK293 and MCF-7 cell growth in a dose- and time- dependent manner (Figure 1A and B). No significant effect was found with 0.1mM sodium butyrate for either cell line; however, increasing concentrations of sodium butyrate increasingly reduced the proliferation over time. The effect appeared more dramatic for MCF-7 cells: at 24 h, no difference could be seen at any concentration for HEK293, whereas for MCF-7 cells, the 2.0 mM (*P*<0.05) and 8.0 mM (*P*<0.01) groups had significantly reduced A_450nm_ values compared with the vehicle control. Additionally, at 48 h with 0.1, 0.5, 2.0 and 8.0 mM sodium butyrate concentrations, the inhibitory rate of HEK293 (5.5%, 17.9%, 22.5%, and 22.5%) was lower than that of MCF-7 cells (7.2%, 20.7%, 43.2%, and 56.5%). These findings suggest that MCF-7 cell growth is inhibited more strongly than HEK293 by sodium butyrate.

To directly compare the effects of sodium butyrate on MCF-7 versus HEK293, we calculated the % viability for 0.5 mM and 4.0 mM sodium butyrate treatment at different times. MCF-7 cells were more greatly inhibited than HEK293 were upon 0.5 mM sodium butyrate treatment for 96 h (72.5% versus 92.0%, *P<*0.01) and upon 4.0 mM sodium butyrate treatment for 24 h (65.7% versus 86.6%, *P<*0.05), 48 h (46.5 versus 65.8% %, *P<*0.05), 72 h (29.5% versus 53.9%, *P<*0.01), and 96 h (26.0% versus 43.1%, *P<*0.01) (Figure 1C). These finding verify that HEK293 cells are more resistant than MCF-7 cells to the cytotoxic effects of sodium butyrate.

### Sodium butyrate decreases the proportion of cells in S phase for both HEK293 and MCF-7 cells

**Figure 2:**
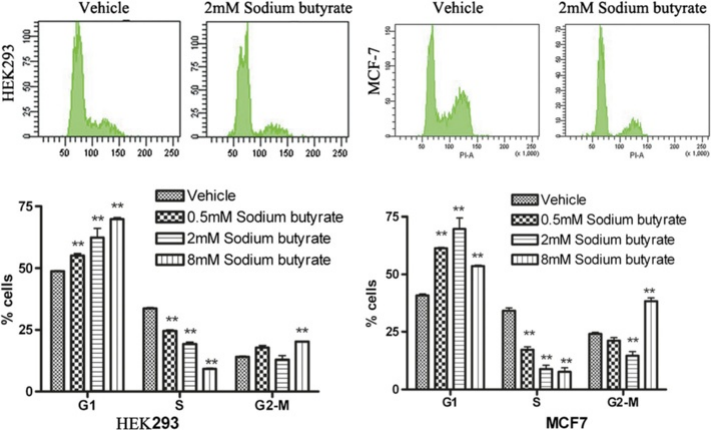
**Sodium butyrate decreases the proportion of cells in S phase for both HEK293 and MCF-7 cells.** HEK293 and MCF-7 cells were treated with DMSO vehicle, 0.5, 2.0, or 8.0 mM sodium butyrate for 24 h. Cell cycle analysis was performed by flow cytometry using propidium iodide staining. Representative histograms are shown above, and quantification of the cells in each phase of the cell cycle is provided below. The values represent the means + SD of triplicate experiments. **P< 0.01 versus vehicle-treated cells.

Cell proliferation is closely associated with the cell cycle, which is regulated by checkpoints that are activated by the DNA damage response pathway. To determine whether the differential effects of sodium butyrate on proliferation in HEK293 and MCF-7 cells can be explained by differential redistribution of cell cycle progression, we treated each cell line for 24 h with 0.5, 2.0, or 8.0 mM butyrate. Our results demonstrate that for both cell lines, sodium butyrate robustly induces the accumulation of cells in G_1_ and G_2_ phase with a concomitant decrease of cells in S phase (Figure 2). These results suggest that sodium butyrate triggers cell cycle checkpoints in both cell lines, indicating that the differences in growth response to sodium butyrate are not caused by differential control of the cell cycle.

### Sodium butyrate suppresses cell growth synergistically with etoposide, and the effect is more dramatic for MCF-7 cells than for HEK293 cells

**Figure 3:**
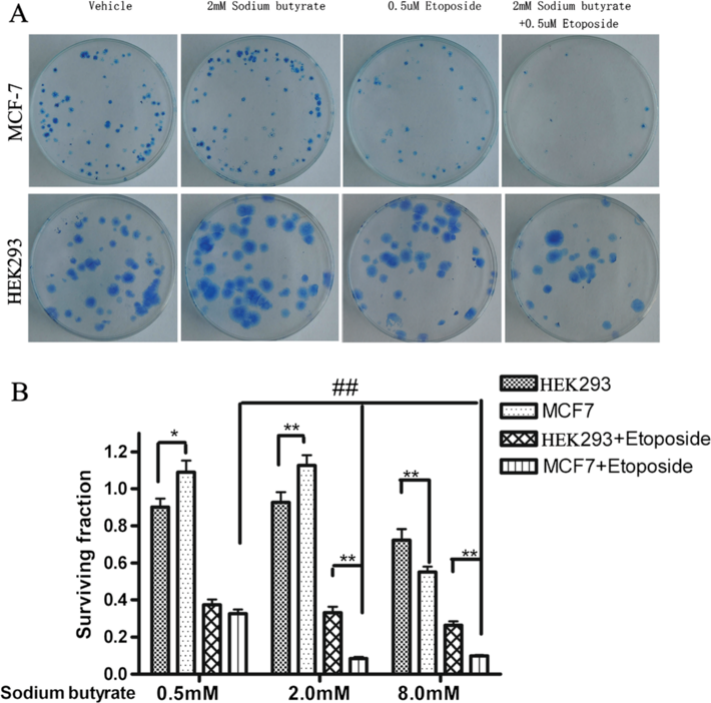
**Sodium butyrate enhances the cytotoxic effects of etoposide, and the effect is more dramatic in MCF-7 cells than in HEK293 cells. (A)** MCF-7 and HEK293 cells were pre-treated with DMSO vehicle or 2 mM sodium butyrate for 4 h, and then were treated with vehicle control or 0.5 μM etoposide for 20 h. After the treatment period, cells were re-seeded for colony forming assay and grown for 15 days. The colonies were stained with 0.5% methylene blue for visualization. Results are representative of 3 independent experiments. **(B)** HEK293 and MCF-7 cells were treated for 4 h with 0, 0.5, 2.0, or 8.0 mM sodium butyrate and then were exposed to DMSO vehicle or 0.5 μM etoposide for 20 h. After the treatment period, cells were re-seeded for colony forming assay. The fraction of surviving colonies was determined for the 0.5, 2.0, and 8.0 mM sodium butyrate groups relative to the corresponding 0 mM sodium butyrate group.*P< 0.05 and **P< 0.01 for MCF-7 versus HEK293 cells, ##*P*< 0.01 versus the corresponding co-treatment of 0.5 mM sodium butyrate plus etoposide in MCF-7 cells. Results represent the means + SD of quadruplicate experiments.

To further verify the growth inhibitory effects of sodium butyrate in HEK293 and MCF-7 cells, an equivalent number of each type of cell were seeded for colony forming assay after 24 h treatment with vehicle or 2.0 mM butyrate in the absence or presence of 0.5 μM etoposide, a classic DNA damage reagent. Because co-treatment with HDACi and Topo II inhibitor only has a synergistic effect if HDACi is administrated before Topo II inhibitor [9], we exposed the cells to 2.0 mM butyrate before etoposide was added. While each drug alone had minimal effect, the two drugs together decreased the number of colonies that grew after 15 days in both HEK293 and MCF-7 cells; however, the inhibitory effects of the two drugs in combination were more obvious for MCF-7 cells (Figure 3A). These results suggest that the two drugs may function synergistically to reduce cell viability and that the synergistic effects are more pronounced for cancerous MCF-7 cells.

To verify these findings, we treated cells with a range of doses of sodium butyrate with or without etoposide for 24 h prior to plating. In the absence of etoposide, the survival fraction for the 0.5 or 2.0 mM sodium butyrate doses, was significantly increased for MCF-7 cells compared to HEK293 cells, but the opposite trend was observed for the 8.0 mM sodium butyrate dose (Figure 3B). Additionally, in the presence of etoposide, 2.0 mM or 8.0 mM sodium butyrate led to more obviously decreased colony formation for MCF-7 cells than for HEK293 cells. These results suggest that under lower, non-toxic doses of butyrate, MCF-7 cells may have intrinsic mechanisms for protection from cell death, but that under harsher drug conditions of either higher dose sodium butyrate or a combination of sodium butyrate and etoposide, cancerous MCF-7 cells are more sensitive than HEK293 cells to drug treatment.

### Sodium butyrate enhances etoposide-induced γ-H2AX accumulation to a greater extent in MCF-7 cells than in HEK293 cells

**Figure 4:**
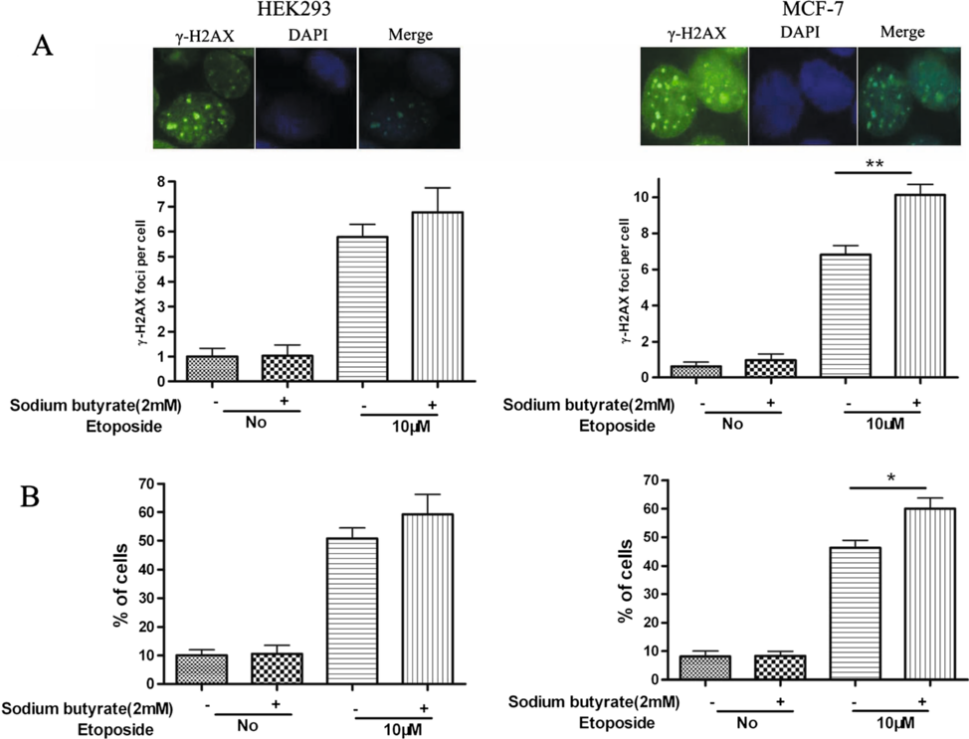
**Sodium butyrate statistically increases γ-H2AX foci induced by etoposide in MCF-7 cells but not HEK293 cells.** HEK293 and MCF-7 cells growing on slides in 24 well plates were exposed to DMSO vehicle or 2.0 mM sodium butyrate for 4 h followed by 20 h DMSO vehicle or 10 μM etoposide treatment. Following the treatment period, cells were fixed and assessed for γ-H2AX foci. **(A)** The average numbers of γ-H2AX foci per cell are shown. The inset shows a representative image of staining for a γ-H2AX-positive cell. **(B)** The % of foci-containing cells with >5 foci was calculated. *P< 0.05 and **P< 0.01. Results represent the mean +SD of 6 samples with 100 to 300 cells counted per slide.

To further investigate the potential contribution of DNA DSBs to the differential growth response of MCF-7 and HEK293 cells, we tested whether sodium butyrate can increase nuclear γ-H2AX foci, a marker of DNA DSBs. 2.0 mM butyrate alone did not significantly affect γ-H2AX foci in either cell line. However, the addition of 10 μM etoposide caused a difference in the number of foci per nucleus, which was statistical in MCF-7 cells, but not in HEK293 cells (Figure 4A, *P<*0.01). Furthermore, the percentage of foci–containing cells was significantly increased in MCF-7, but not HEK293 cells (Figure 4B, *P<*0.05). These results suggest that low dose butyrate treatment can heighten etoposide-induced DNA damage and γ-H2AX accumulation to a greater extent in MCF-7 cells than in HEK293 cells.

### Sodium butyrate suppresses nuclear expression of DNA DSB repair proteins induced by etoposide in MCF-7 and HEK293 cells

**Figure 5:**
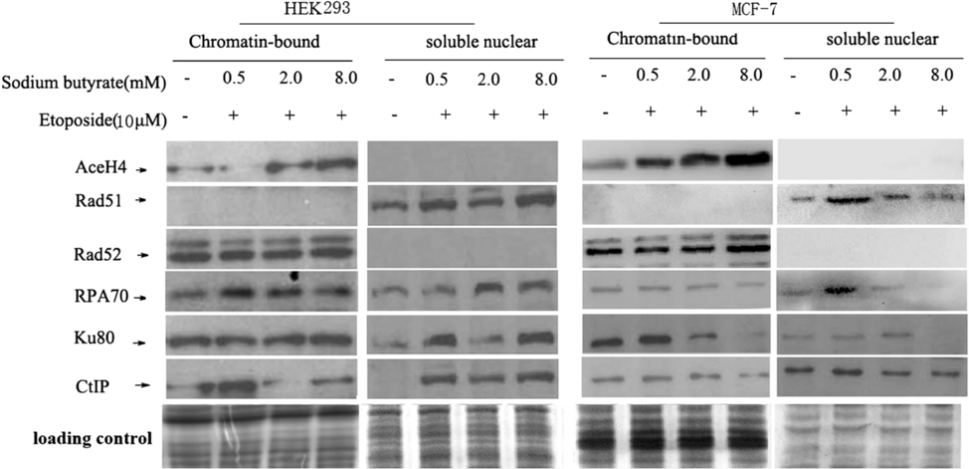
**Sodium butyrate modulates the nuclear expression of DSB repair proteins induced by etoposide in MCF-7 and MEL293 cells.** HEK293 and MCF-7 cells were pre-treated with DMSO vehicle or 0.5, 2.0, 8.0 mM sodium butyrate for 4 h before exposure to DMSO vehicle or 10 μM etoposide for 20 h. After the treatment period, the cells were harvested, and then soluble nuclear protein and chromatin bound protein were extracted using a subcellular protein fraction kit. 50 μg proteins were loaded for western blot analysis. Coomassie blue staining gel is shown as loading control. Results are representative of three independent experiments.

To determine how sodium butyrate may affect the etoposide-induced DSB repair process, we examined the expression of proteins involved in the DNA damage response pathway. Because DSBs are generated in DNA, which is located in the nucleus and is characterized by surrounding chromatin architecture, we assessed the expression changes in chromatin-bound and nuclear soluble protein fractions. HEK293 and MCF-7 cells were pre-treated cells with sodium butyrate before exposure to 10 μM etoposide for inducing detectable DSBs and DNA damage response. As shown in Figure 5, the chromatin-bound protein of Acetyl-histone H4 (AceH4) is increased in both HEK293 and MCF-7 cells; however, the increase in MCF-7 cells is observed at a lower sodium butyrate dose. This is consistent with the greater sensitivity of MCF-7 cells as compared to HEK293 cells to the sodium butyrate/etoposide combination.

The results for the other proteins we tested are more complex. Chromatin-bound Rad52 did not show reproducible modulation upon treatment with sodium butyrate and etoposide. However, RPA70, Ku80 and CtIP showed variable patterns of modulation, with increase in chromatin-bound protein in HEK293 cells at certain doses of sodium butyrate, but decrease gradually in MCF-7 cells follow increasing doses. The soluble nuclear portion of these proteins also showed variable trends of modulation, high doses (2 mM and 8 mM) of sodium butyrate attenuate the upregulation of Rad51, RPA70 and CtIP induced by etoposide in MCF-7 cells but not in HEK293 cells. Collectively, these data support the idea that the HDACi sodium butyrate is involved in the mechanism of etoposide-induced DSB damage and repair to improve its anticancer activity.

## Discussion

Research has shown that HDACi’s affect gene transcription, induction of cell cycle arrest, differentiation, apoptosis, and inhibition of cell survival, particularly in tumor cell lines [4,10]. Consistent with those data, we found that the growth of both HEK293 cells and cancerous MCF-7 cells are inhibited in a dose- and time-dependent manner by sodium butyrate treatment alone. Core histone acetylation and deacetylation are connected with checkpoint activation and repression [11], and HDACi also mediates acetylation-dependent changes in non-histone proteins involved in cell cycle regulation [12]. Our data show that the cell cycle of both HEK293 cells and cancer MCF-7 cells are arrested by sodium butyrate mostly in G1 but also in G2 phases and reduced in S phase simultaneously, and a similar result has been observed in Hela cells before [13]. So, cell cycle arrest might be one of the main reasons for the effects of sodium butyrate in promoting inhibition of proliferation.

HDAC_1/2_ play redundant and essential roles in tumor cell survival. Their deletion leads to nuclear bridging, nuclear fragmentation, and mitotic catastrophe, mirroring the cytotoxic effects of HDACi’s on cancer cells [14]. Human HDAC_1/2_ function in the DNA damage response pathway to promote DNA repair, and their deletion leads to hypersensitivity to DNA-damaging agents and sustained DNA-damage signaling [15]. Consequently, various HDACi’s with different HDAC inhibition profiles have been reported to induce DNA damage [1,5,16]. Sodium butyrate exhibits radiosensitizing effects in several cancers, such as cervical cancer and melanoma cells [17]. We demonstrated that low dose and short times of sodium butyrate incubation led to protection from killing in MCF-7 cells, as measured by colony forming assay, whereas higher doses or a combination of sodium butyrate and etoposide had clear effects on the viability of MCF-7 cells. Higher concentrations of HDACi are believed to exert cell cycle redistribution, induction of apoptosis, and downregulation of surviving signals; whereas lower, non-toxic doses of HDACi might not be strong enough to produce DSBs, but might allow gene activation that sustains cell survival in MCF-7 cells. Consistently, 24 h treatment of 2.0 mM sodium butyrate alone did not affect cell viability, with no detectable increase in the γ-H2AX foci in either HEK293 or cancer MCF-7 cells line; however, when combined with etoposide, 2.0 mM butyrate sensitized MCF-7 cancer cells but not HEK293 cells to produce more γ-H2AX foci. This chemotoxic synergy is likely due to both increased numbers of DSBs and hyperactivation of the cytotoxic arm of the DNA damage response.

HDACi’s have been shown to directly downregulate homologous recombination and non-homologous end-joining in many cancer cell lines [17-20]. For example, in prostate cells, treatment with HDACi downregulates the protein expression levels of BRCA1, RAD51 and DNA-PK, and the mRNA expression levels of ATM, BRCA1, BRCA2, RAD51 and XRCC4 [4,21]. Synchronized HeLa cells in G1 phase have decreased non-homologous end-joining in the presence of butyrate [17]. Additionally, HDACi’s attenuate the upregulation of Ku70, Ku80 and DNA-PK induced by ionizing radiation in several cancer cells [22,23]. On the other hand, the effects of HDACi on non-homologous end-joining and homologous recombination proteins to date have not been observed in normal human cells.

Interactions between chromatin and DNA damage response proteins are central to the cellular response to DSBs. Assembly factors and repair at most DNA breaks in mammalian cells occurs by recruiting complexes from the nucleoplasm. Therefore, the protein expression of chromatin-bound and soluble nuclear compartments reflects an underlying state of DSB repair. We found that sodium butyrate differentially affects the nuclear expression of some DSB repair proteins in MCF-7 and HEK293 cells. The chromatin-bound protein of Acetyl-histone H4 was more highly induced by sodium butyrate in the presence of etoposide for MCF-7 cells than for HEK293 cells. Differences were also observed for Rad51 (a key component of homologous recombination pathway, which resides only in the soluble nuclear fraction), Ku80 (which serves as a key initiator component of the non-homologous end-joining pathway), CtIP (which initiates DSB end resection and generates 3’ ended single strand DNA overhangs necessary for homologous recombination), and RPA70 (which is coated onto the overhangs of single stranded DNA created during resection). However, Rad52 (which facilitates upstream and downstream sequences of DSB annealing during signal -strand annealing pathway) did not consistently change with increasing doses of sodium butyrate. These results indicate that the DSB repair pathway induced by etoposide is suppressed after butyrate pre-treatment in both cell lines, but that the effect is more dramatic for cancer MCF-7 cells. These results suggest that sodium butyrate may enhance cancerous cell MCF-7 killing by inhibiting recruitment of repair factors to damaged DNA and reducing its repair capacity.

We have compared two different cell lines, one cancerous and one non-cancerous; however, we acknowledge that these cell lines also have differences in the species and tissue derivation. Therefore, further work is needed to determine whether the differences in response that we have observed in this study may extend to other cancerous and non-cancerous cell pairs. Undoubtedly, there is a range of response to sodium butyrate that is likely to vary according to the cancer type and stage of malignancy. However, our results provide important mechanistic information about how sodium butyrate may function differentially in different cells, which may explain the therapeutic efficacy and low toxicity of HDACi’s in selectively killing cancer cells [3-5]. Practically, the combination of sodium butyrate may enhance the efficacy of etoposide and permit lower concentrations. Additionally, the understanding of how these two drugs function synergistically may facilitate the development of future therapeutics to selectively treat cancer.

## Conclusions

In summary, our findings indicate that the cytotoxic effects of the HDACi sodium butyrate occur, in part, via down-regulation of DSB repair protein accessibility to the nucleus and the sites of damage, with the outcome of affecting the repair capacity. Differences in the chromatin compaction in HEK293 cells and cancer MCF-7 cells in response to HDACi treatment might determine distinct fates between survival and death by controlling the DSB repair pathway. Consequently, sodium butyrate sensitizes cancerous MCF-7 more highly than HEK293 cells to the cytotoxic effect of DNA damage agents. Further work is needed to elucidate the precise pathways and targets by which HDACi’s exert these chemosensitizing effects in cancer cells.

### Availability of supporting data

The data set supporting the results of this article is included within the article (and its additional file(s)).

## Abbreviations

HEK293: Human embryonic kidney 293
HDACi: Histone deacetylase inhibitor
HDAC: Histone deacetylase
γ-H2AX: Histone variant H2A phosphorylated at serine 139
DSBs: Double strand breaks
DNA-PK: DNA-dependent Protein Kinase
CtIP: C-terminal binding protein-interacting protein
ATM: Ataxia telangiectasia mutated
RPA: Replication protein A
BRCA1: Breast cancer 1
BRCA2: Breast cancer 2
XRCC4: X-ray repair cross complementing4
Topo II: Topoisomerase II
CCK-8: Cell Counting Kit
TBST: TBS containing 0.1% Tween20
